# Effective anti-cancer property of *Pouteria sapota* leaf on breast cancer cell lines

**DOI:** 10.1016/j.bbrep.2018.06.004

**Published:** 2018-06-28

**Authors:** D. Sathya Prabhu, A. Panneer Selvam, V. Devi Rajeswari

**Affiliations:** a Department of Biomedical sciences, School of Biosciences and Technology, VIT University, Vellore 14, Tamil Nadu, India; b Department of Zoology, Thiruvalluvar University, Serkadu, Vellore 14, Tamil Nadu, India

**Keywords:** *Pouteria sapota*, Anti-cancer activity, Anti-oxidants, *In vitro*, MCF-7

## Abstract

Natural products are vital in drug discovery and the search for anticancer agents has been significant importance to the researchers for a long time. In the present study, aqueous leaf extract of *Pouteria sapota (P.sapota)* was evaluated for its cytotoxic activity. The leaf extract was preliminarily screened for antioxidant activity using DPPH method for Radical Scavenging Activity, Hydrogen Peroxide Scavenging Activity and Reducing Power Activity. Further, the aqueous leaf extract was screened for cytotoxic activity against breast cancer cell lines (MCF-7) in vitro. The results of the study showed that aqueous extract of the *P.sapota* leaf was rich in phytochemicals, antioxidant activity and showed a significant anti-cancer activity against tested MCF-7 cell lines. The present study was designed to evaluate the anticancer potential of *P.sapota* leaf. The antioxidants present in *P.sapota* have strong cytotoxic activity suggests that it can be considered for anti-cancer treatment.

## Highlights

• The *P. sapota* showed a significant anti-cancer activity against cell lines.
• The GC analysis showed many active constituents.
• The active constituents exhibit biological activity.

## Graphical abstract

**Figure:**
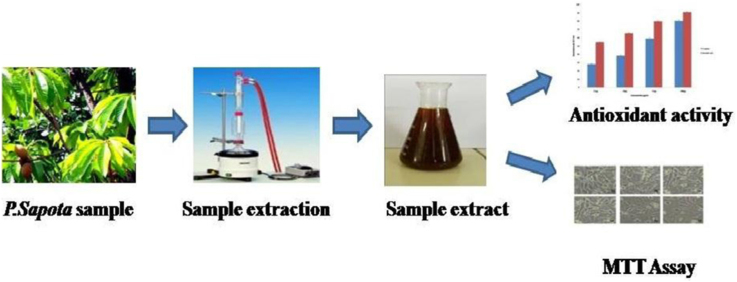

## 1 Introduction

Medicinal plants are effective anticancer agents since centuries [1]. Different parts of medicinal plants were investigated in order to find out its anti-cancer agents [2]. The anti-oxidants present in the medicinal plants was possibly responsible for the anticancer activity [3]. Likewise, *P.sapota* is a well-known fruit crop not majorly investigated for its medicinal and biological properties. This plant is also known as “mamay” in native Central America, Mexico and in many parts of the world; the plant was majorly grown for its fruits, which are enriched with abundant of nutrients [4]. The leaf extract was found to be effective biologically against blowfly [5]. However, the parts of the plants were not deeply studied for its biological activity. Hence, the present study was designed to find the in-vitro antioxidant and cytotoxic activity of *P.sapota* leaf aqueous extract.

## 2 Materials and methods

### 2.1 Chemicals

All the chemicals used for this study are of analytical grade and were purchased from Sigma Aldrich, USA; Roche, Germany; and SD Fine Chemicals, India.

### 2.2 Sample collection

The fresh leaves of *P.sapota* (500 g) were collected from Botanical Garden of VIT University, Vellore, Tamil Nadu, India. Leaf samples were identified by taxonomist at Department of Biological Sciences, VIT University. A voucher specimen was deposited at VIT plant repository for further reference. Immediately after collection, leaves were cleaned with distilled water extensively, wiped with sterile cotton and shade dried under room temperature. Air dried plant leaves were then pulverized into fine powder mechanically and stored at − 20 °C until use.

### 2.3 Preparation of extract

Aqueous extract of *P.sapota* was carried out by adopting the previous methodology with slight modification [6]. In brief 500 g of pulverized *P.sapota* leaf powder was soaked in 250 ml of distilled water at room temperature (26 ± 1 °C) for 48 h under continuous orbital shaking (125 rpm). The resultant mixture was then filtered and concentrated by lyophilizer. The lyophilized aqueous extract weighing 26.3 g was used for further biological assay experiment.

### 2.4 Antioxidant Assays

#### 2.4.1 DPPH method for radical scavenging activity

The radical scavenging activity of *P.sopata* leaf extract was estimated using DPPH method [7], [8], [9]. In this assay, 0.1 mM solution of DPPH in methanol was prepared and 1 ml of this solution was added to 3 ml of the aqueous extracts of the sample at different concentrations (25, 50, 75 and 100 µg/ml) with the standard ascorbic acid. These mixtures were shaken vigorously and allowed to stand at room temperature for 30 min. The absorbance was measured at 517 nm using UV–VIS spectrophotometer and the results obtained were inversely proportional to radical scavenging activity.

The percentage (%) of radical scavenging activity is measured by the formula

$$\%\;\mathrm{of}\;\mathrm{scavenging}\;\mathrm{activity}=(1-A_{1}/A_{0})\times 100$$

where, A_1_ is OD of test sample and A_0_ is OD of control

#### 2.4.2 Reducing power activity

The reducing power activity of *P.sopata* leaf extract was estimated using standard method [10], [11]. In this method, 0.1 ml of the leaf extract of different concentrations (25, 50, 75 and 100 µg/ml) was mixed with 2.5 ml of phosphate buffer (pH 6.6) and 2.5 ml of 1% potassium ferric cyanide respectively. All tubes were incubated at 50 °C for 20 min and after incubation 2.5 ml of 10% trichloroacetic acid was added to each test tube. Then the tubes were centrifuged at 10,000 rpm for 10 min, to the 5 ml supernatant (upper layer) of the centrifuged samples 5 ml distilled water was added and mixed well. To the prepared 10 ml of samples 1 ml of 0.1% ferric chloride was added correspondingly in each tube. Finally, the absorbance of each sample was measured at 700 nm against a blank (distilled water).

The percentage inhibition was calculated by the equation.

$$\%\;\mathrm{inhibition}=(1-A_{1}/A_{0})\times 100$$

where, A_1_ is OD of test sample and A_0_ is OD of control

#### 2.4.3 Hydrogen peroxide Scavenging Activity

The Hydrogen peroxide Scavenging Activity was determined according to the standard method [12], [13]. In this method, 1 ml of the sample in different concentrations of (25, 50, 75 and 100 µg/ml) was mixed with 2 ml hydrogen peroxide solution respectively. These tubes were incubated at room temperature for 10 min. After incubation absorbance of the samples were checked at 230 nm in a spectrophotometer.

The percentage inhibition was calculated by the equation

$$\%\;\mathrm{inhibition}=(1-A_{1}/A_{0})\times 100$$

where, A_1_ is OD of test sample and A_0_ is OD of control

### 2.5 MTT assay

The human breast cancer cell lines (MCF-7) obtained from National Centre for Cell Sciences (NCCS), Pune, India was used for MTT (*(3-(4, 5-dimethylthiazol-2-yl)-2, 5-diphenyltetrzolim bromide*) assay [14], [15]. The MCF-7 cell lines were grown and maintained in MEM medium in a 5% CO_2_ incubator at 37 °C respectively. Further, the cell suspension was harvested by centrifugation and the adherent cells were released from their substrate by trypsinization or scraping. Later the cells were resuspended in the medium at a quantity of 1 × 10^6^ per ml. Further, a serial dilution was done to dilute the cells from 1 × 10^6^ to 1 × 10^3^ cells per ml respectively. Thereby, 0.1 ml of the above dilutions was plated out into the wells of a microtiter plate and control was maintained with medium alone. The cells were incubated for 12 h at 37 °C and 0.01 ml of MTT reagent (prior to the experiment MTT was dissolved in phosphate buffered saline (pH 7.4) and stored at 4 °C) was added to each well including controls and incubated for another 4 h correspondingly. Then the cells were periodically viewed under a microscope for the presence of intracellular punctuates purple precipitate. When the purple precipitate was clearly visible 0.1 ml of solubilization solution [It is a combination of 40% dimethylformamide in 2% glacial acetic acid mixed with 16% SDS (pH 4.7) and stored at room temperature] was added to all the wells, including controls and mixed well without shaking. The absorbance was recorded at 570 nm and the percentage of cell viability is calculated by using the following equation.

%ofcellviability=(1−(ODoftest/ODofcontrol))×100

## 3 Results

### 3.1 Antioxidant assays

**Fig. 1:**
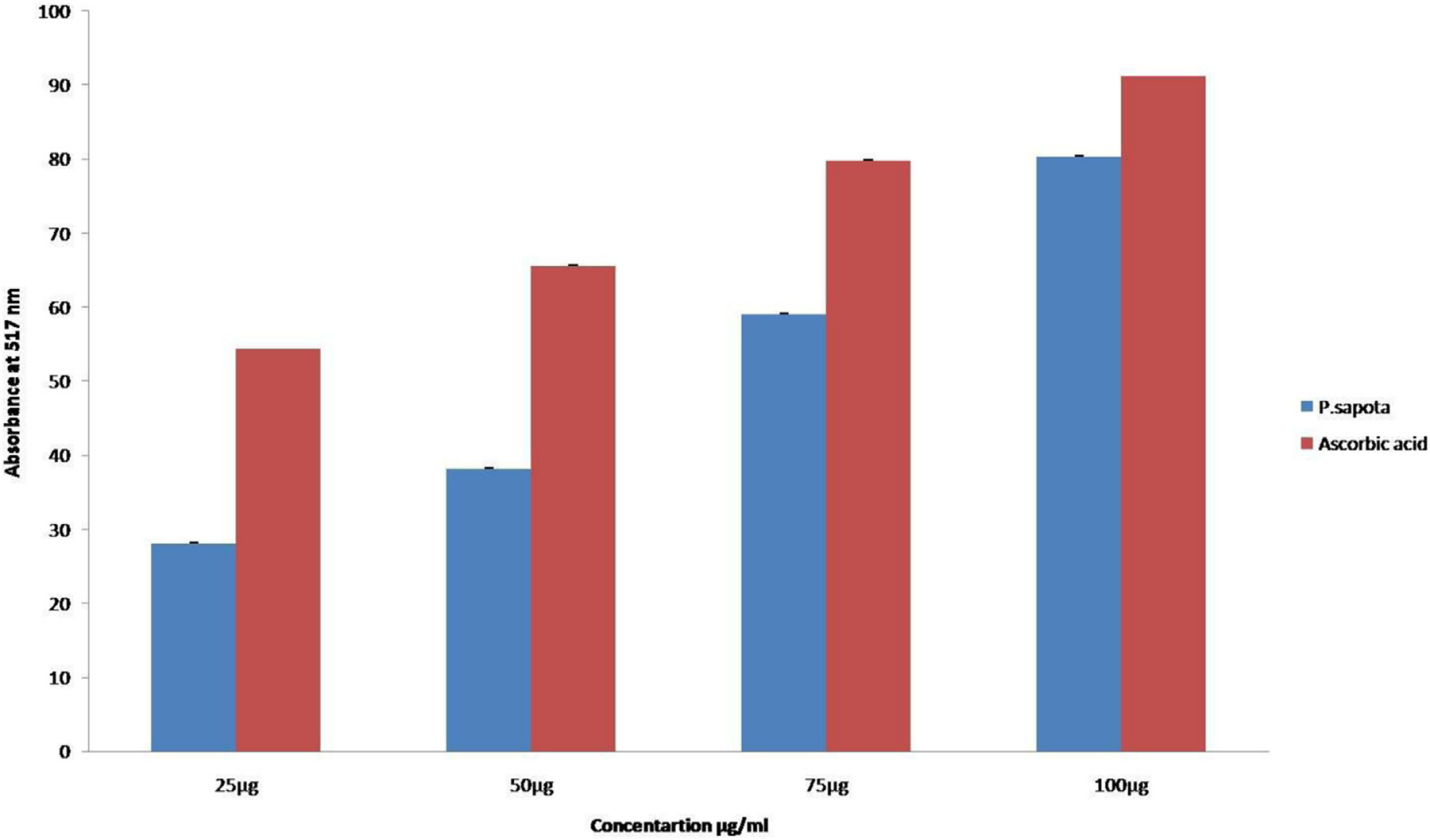
The graphical representation of the DPPH antioxidant activity of *P.sapota* leaf extract with the standard ascorbic acid.

**Fig. 2:**
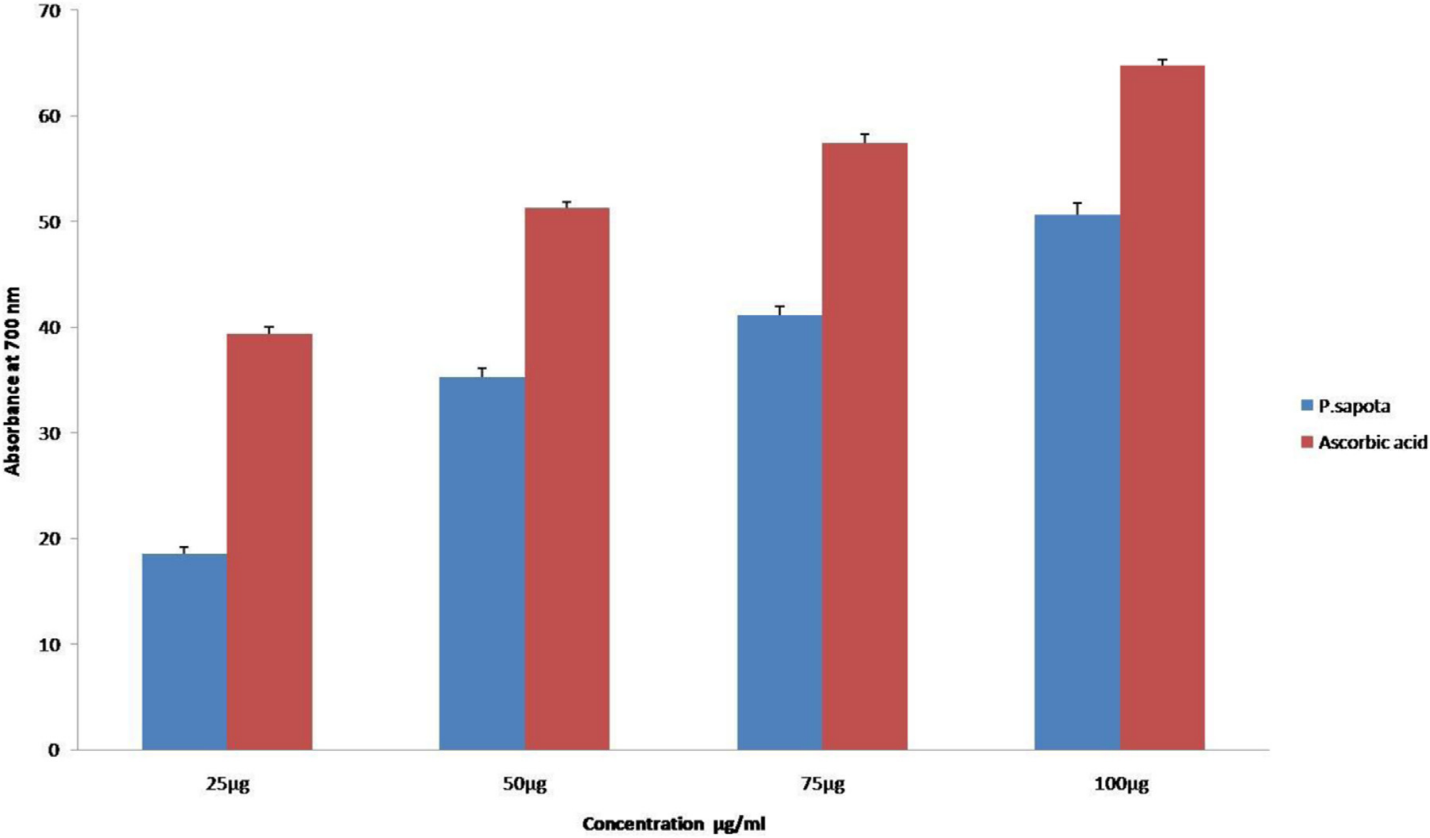
The graphical representation of the Ferric acid reducing assay of *P.sapota* leaf extract with the standard ascorbic acid.

**Fig. 3:**
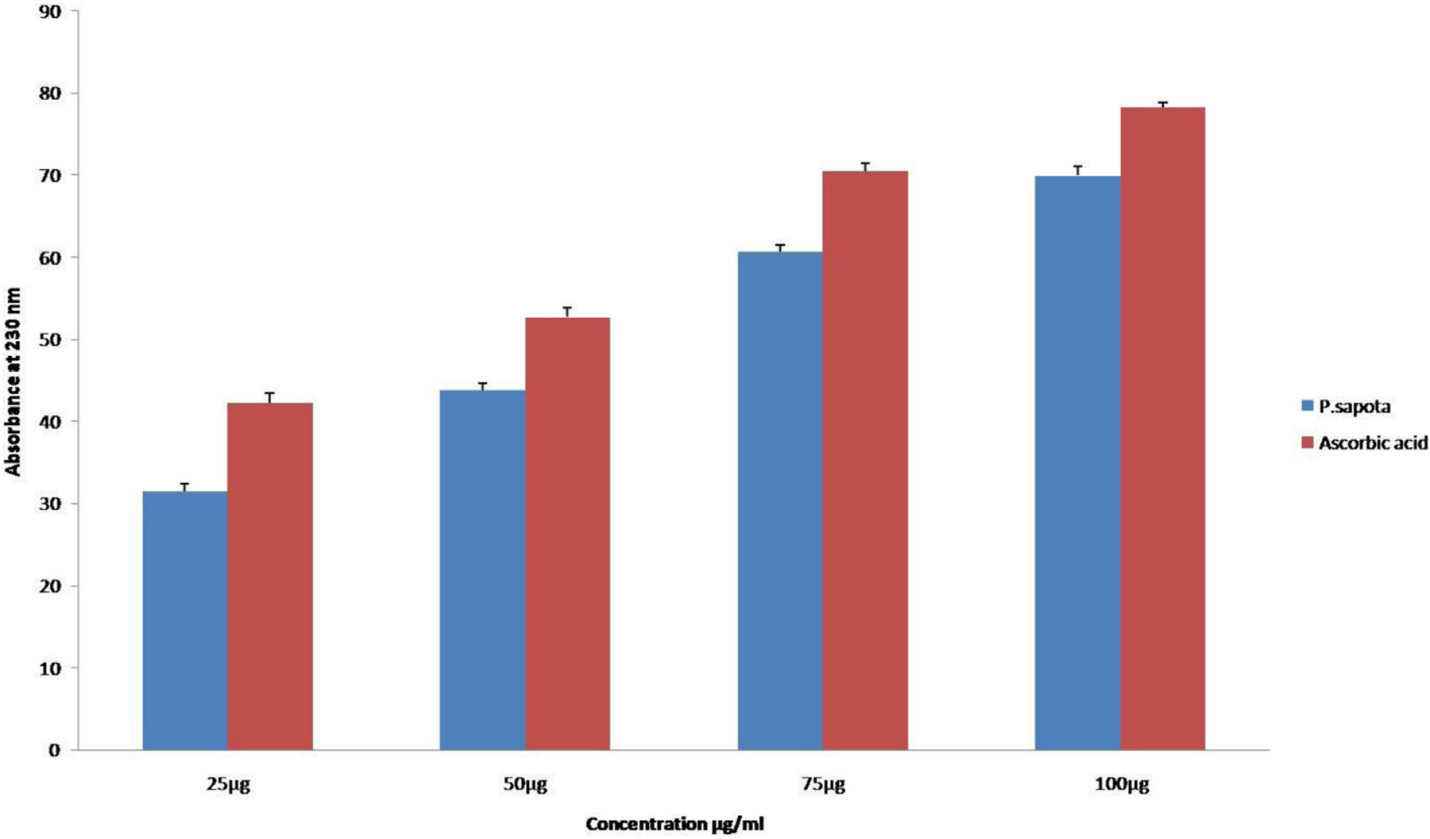
The graphical representation of the hydrogen peroxide radical scavenging assay of *P.sapota* leaf extract with the standard ascorbic acid.

The DPPH assay shows the presence of antioxidant capacity among the concentrations of the sample simultaneously. The antioxidant activity showed an equivalent strength to that of the standard, ascorbic acid comparatively (Fig. 1). The antioxidants present in the *P.sopata* leaf extract reduce ferric cyanide to ferrous compound with a strong reducing power capacity at all the concentrations (25 µg, 50 µg, 75 µg and 100 µg) respectively (Fig. 2). The leaf extract of *P.sopata* showed a strong ability to scavenge hydrogen peroxide with the standard ascorbic acid (Fig. 3). The antioxidants present in the leaf extract were responsible for the scavenging activity, thus all the concentrations of the sample (25 µg, 50 µg, 75 µg, and 100 µg) showed inhibitory action for the production of free hydroxyl radicals.

### 3.2 MTT Assay

**Fig. 4:**
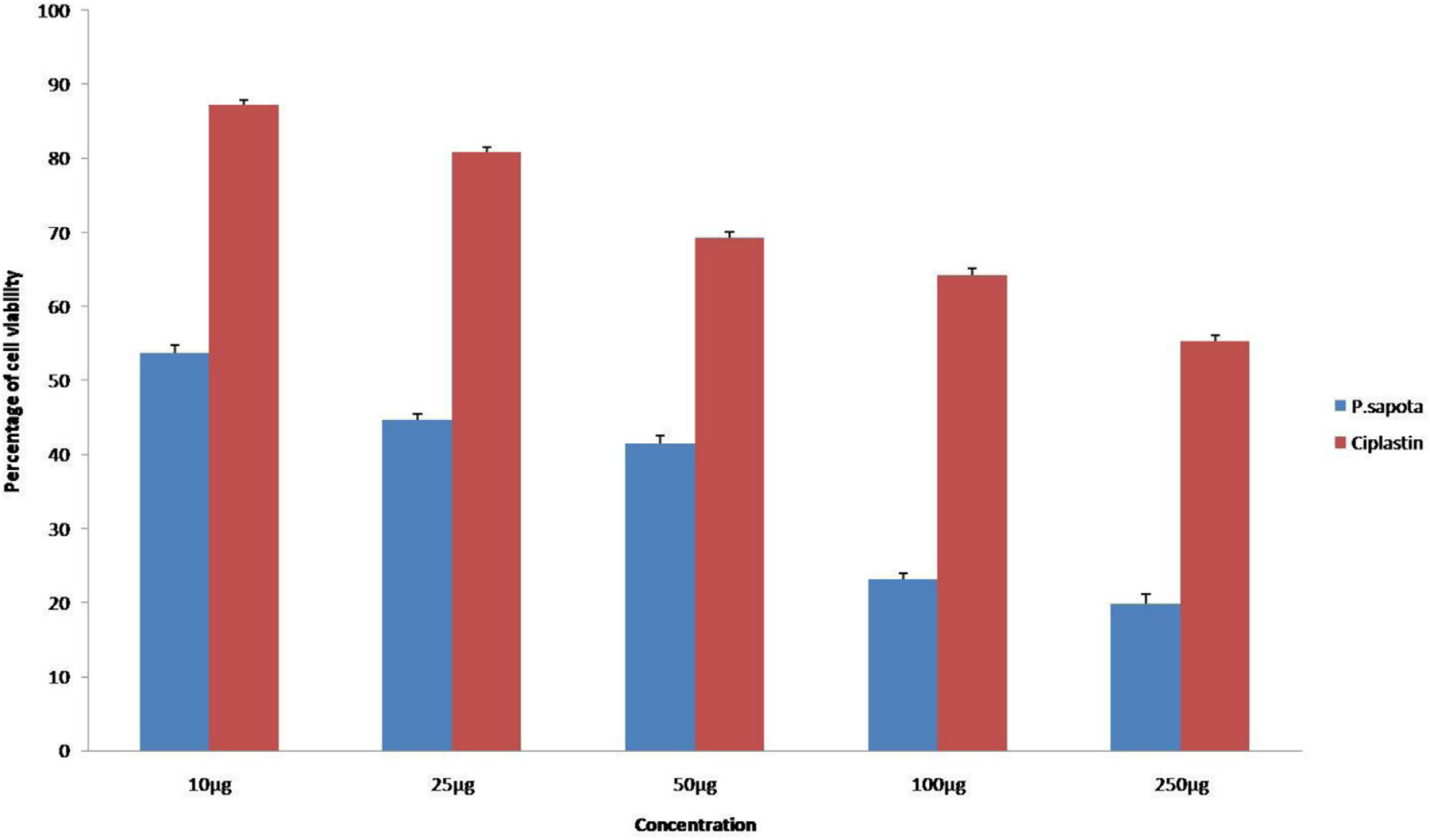
The quantitative comparison of cytotoxicity effect of *P.sopata* leaf extract with the standard Ciplastin.

**Fig. 5:**
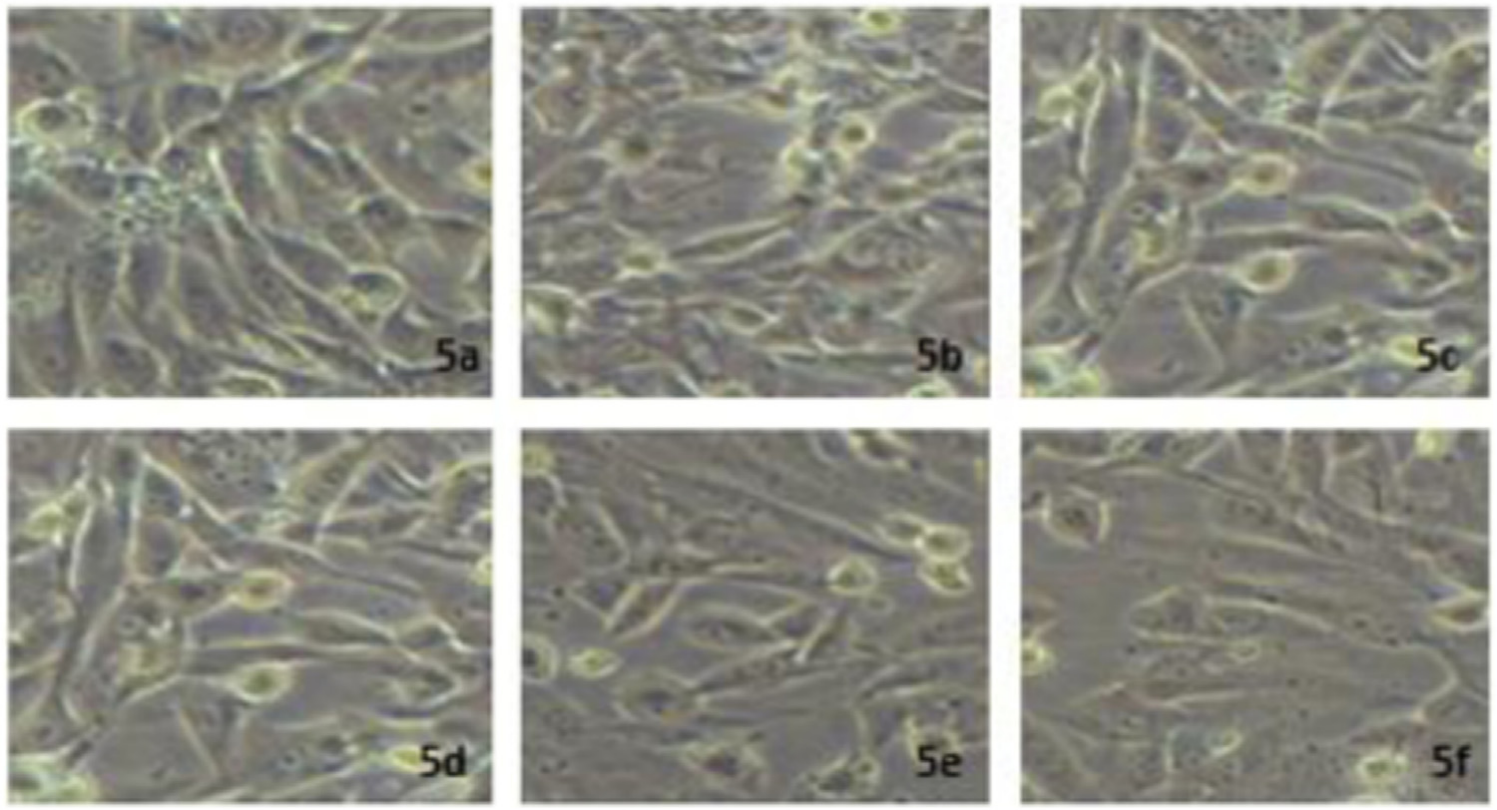
MTT images show the effect of P.sapota leaf extract on breast cancer cell line MCF-7 at different concentarions with ciplastin as the control (5a) Control; 5b) 10 µg; 5c) 25 µg; 5d) 50 µg; 5e) 100 µg; 5 F) 250 µg).

Using MTT assay, the effect of *P.sapota* leaf extract on breast cancer cell lines (MCF-7) cell proliferation was evaluated [16]. The assay has exposed the cytotoxic effect of leaf extract on cancer cells with cisplatin as the control (Fig. 4) apparently inducing its cell proliferation. The results showed changes in the cell morphology in all concentrations (25 µg–125 µg) (Fig. 5).

## 4 Discussion

**Fig. 6:**
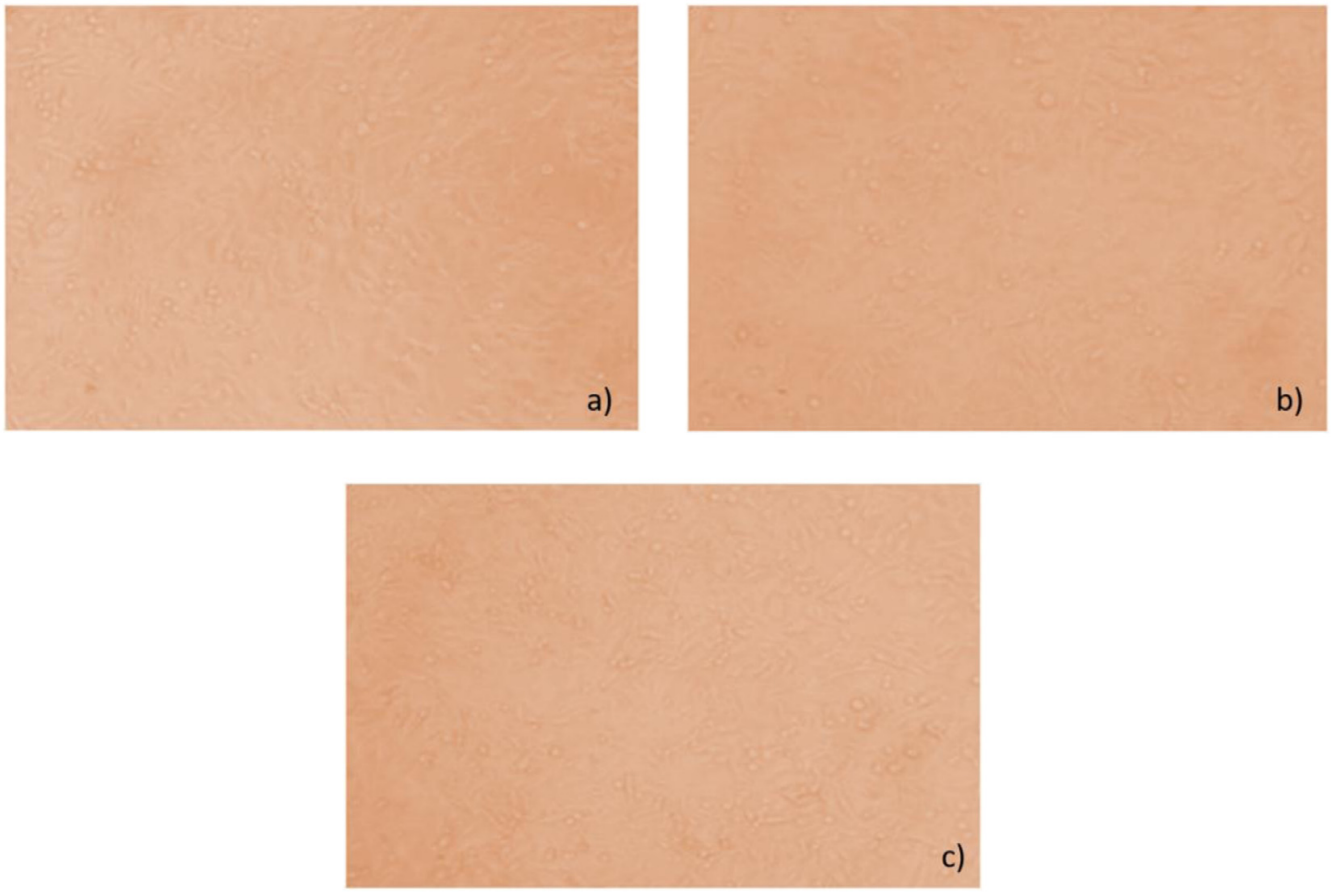
MTT images show the effect of P.sapota leaf extract on breast cancer cell line MDAMB-231 at different concentarions with ciplastin as the control (6a) Control; 6b) 10 µg; 6c) 320 µg).

Antioxidants are capable of either suppressing or inhibiting the oxidation processes that are occurring in the presence of atmospheric oxygen or any reactive oxygen species [17]. DPPH assay was the most reliable antioxidant assay to determine the antioxidants present in medicinal plants [18], [19], [20]. So far, the antioxidant capacity of *P.sapota* leaf has been not reported in the literature. This study found the strong antioxidant activity using DPPH assay in four different concentrations (25 µg, 50 µg, 75 µg, and 100 µg). The results were similar to the antioxidant activity results that were reported in earlier studies [21], [22], [23], [24]. In addition to this, the reducing power activity directly reflects the antioxidant capacity of the sample [25], [26]. The reducing power activity of *P.sopata* leaf extract was due to the presence of antioxidants that are capable to break the free radicals by donating its hydrogen atom and rapid decomposition of hydrogen peroxide into oxygen resulting in the neutralization of water [10]. Further, the hydrogen peroxide scavenging activity confirms the ability of antioxidants present in the *P.sapota* leaf extract, where decreasing the levels of prooxidants was noticed [27], [28]. The resultant inhibitory activities are shown by *P. sapota* leaf extract in hydrogen peroxide scavenging assay increases along with the concentration of the extract. Which means the antioxidant activity depends on the concentration of the extract. Fig. 6

Once the presence of rich antioxidants was confirmed using the antioxidant assays, anticancer activity was evaluated using MTT assay. MTT assay is a commonly used calorimetric assay to evaluate the metabolic activity of the cells [29]. This assay measures the cytotoxic activity caused by leaf extract under optimum conditions, at the end of the assay, the purple color product was formed due to the enzymatic reduction of tetrazolium dye to an insoluble form, formazan [30], [31]. So far, quercetin, a bioactive compound was isolated from *P.sapota* fruit and its anticancer activity was proved in cancer cell lines, but not from the leaves [32]. Similarly, many medicinal plants were evaluated for anticancer activity against breast cancer cell lines (MCF-7) [33], [34], [35], [36], [37], [38]. The present study reveals the potency of antioxidant activity increases with its concentration. The antioxidant activity of the leaf extract was believed to be the primarily responsible for cytotoxicity activity against the cancer lines. This procasts a new perception for the leaf extract and it can be used as an efficient adjuvant in the treatment of cancer.

## 5 Conclusion

The work aimed at studying the in vitro antioxidant and cytotoxic activity of *P.sapota* aqueous extract. The antioxidant extract showed cytotoxic activity at different concentrations against the cancer cell lines (MCF-7) with the standards comparatively. This allows for a new perspective of its use in a situation that involves oxidative stress and cell proliferation. In future, the extract can be formulated and it can be used in the treatment of cancer. However, in vivo experiments should be carried out for a better understanding of the mechanism.
