# Supplementary material for: Effective anti-cancer property of Pouteria sapota leaf on breast cancer cell lines
Source: Biochem Biophys Rep. 2018 Jun 28;15:39–44. doi: 10.1016/j.bbrep.2018.06.004 (PMC6039893; doi:10.1016/j.bbrep.2018.06.004)
Supplement: Supplementary file 1 — Supplementary material [file mmc1.docx]

**Conflict of Interest**

The authors declare that they have no competing interests.
